# Antibiotic Resistance Changes in Gram-Positive Bacteria from Urine Cultures: Development Analysis in a Health Area of South-East Spain

**DOI:** 10.3390/antibiotics12071133

**Published:** 2023-06-30

**Authors:** Luis Fernández-Espigares, Itahisa Hernández-Chico, Manuela Expósito-Ruiz, Antonio Rosales-Castillo, José María Navarro-Marí, José Gutiérrez-Fernández

**Affiliations:** 1 Department of Microbiology, School of Medicine, University of Granada-Instituto de Investigación Biosanitaria de Granada (Ibs.GRANADA), 18014 Granada, Spain; luis.fernandez.espigares.sspa@juntadeandalucia.es (L.F.-E.);; 2 Unit of Biostatistics, Department of Statistics, School of Medicine, University of Granada, 18016 Granada, Spain; mexpositoruiz@ugr.es; 3 Program in Clinical Medicine and Public Health, University of Granada & Ibs.GRANADA, 18016 Granada, Spain; 4 Department of Microbiology, Hospital Universitario Virgen de las Nieves-Ibs, 18014 Granada, Spain; josem.navarro.sspa@juntadeandalucia.es

**Keywords:** significant bacteriuria, gram-positive bacteria, antibiotic resistances, urinary tract infection, *Enterococcus*, *Staphylococcus*, *Streptococcus*

## Abstract

This study analyzed the epidemiology and antibiotic susceptibility profile of significant bacteriuria and assessed the impact of adopting EUCAST criteria on antibiotic resistances. A systematic review was performed on publications in English or Spanish between 1 January 2010 and 30 June 2021 on the susceptibility of Gram-positive bacteria isolated in urinary samples in Europe. A retrospective descriptive study was also conducted on the results of 21,838 urine cultures with presumptive urinary tract infection (UTI) obtained during the past five years by the Department of Microbiology of the Virgen de las Nieves University Hospital (Granada, Spain). The activity of various antibiotics was determined, differentiated among various populations, and interpretations compared according to the application of EUCAST or CLSI criteria. Among 21,838 cases of significant bacteriuria, 27.69% were by Gram-positive bacteria, which were *Enterococcus faecalis* in 19.04% and *Enterococcus faecium* in 3.92%. The susceptibility profile remained stable for most antibiotics except for levofloxacin for *E. faecalis* and *Staphylococcus aureus* and nitrofurantoin for *E. faecium*. The resistance of *Enterococcus* spp. and *Staphylococcus* spp. to glycopeptides was exceptionally low in our setting. No significant difference in the prevalence of methicillin-resistant *Staphylococcus aureus* was observed between hospital (26.67%) and community (28.85%) samples. Resistances in our local setting remain stable and appear to be lower than reported in other studies. The adoption of EUCAST vs. CLSI criteria did not produce a general change in resistance rates. Findings suggest the need to revise certain empirical criteria, such as aminoglycoside synergy for *Enterococcus* and for community-origin *S. aureus*.

## 1. Introduction

Gram-positive microorganisms are generally less frequent in urinary tract infections (UTIs). However, they should be considered as possible agents, especially if they are multidrug resistant. This is the case of vancomycin-resistant *Enterococcus faecium* or methicillin-resistant *Staphylococcus aureus* (MRSA) [1,2], whose susceptibility profiles require strict surveillance. Other Gram-positive microorganisms have recently been identified as UTI-producing agents [3,4].

Few data are available on antibiotic resistance to Gram-positive microorganisms in patients with UTI in Spanish hospitals. Studies have habitually centered on Gram-negative bacteria, the most frequent cause of UTI. There is less information on the antibiotic susceptibility of *Enterococcus* spp., commonly isolated in cases of UTI suspicion, or of other emerging microorganisms that can be very difficult to treat. Descriptive studies are useful to compare current resistance patterns with previous observations and those in other settings. Data on antibiotic resistance in local settings can support the optimal selection of empirical therapy for specific patient profiles. These data can also assist in evaluating the reasons for any failure of preventive measures and for any increase in hospital care-related infections.

Over the past few years, many Spanish hospital laboratories have adopted the criteria of the European committee on antimicrobial susceptibility testing (EUCAST) to define minimum inhibitory concentration (MIC) breakpoints for clinical resistance. They had previously followed the criteria of the American Clinical and Laboratory Standard Institute (CLSI) for this purpose. Comparison of the resistance data obtained using each set of criteria may be useful in the development of a consensus on the most appropriate and clinically relevant breakpoints [5,6].

Despite being a viral infection, the COVID-19 pandemic that started in 2019 led to the antibiotic overtreatment of many patients as well as impacting on lifestyles and preventive measures in hospitals and the community, which might have exerted an influence on antibiotic resistances [7]. A descriptive study was conducted on significant bacteriuria (SB) by Gram-positive bacteria in our local setting. The aim was to explore resistance rates in different population groups and to evaluate the impact of applying EUCAST rather than CLSI breakpoint criteria. A systematic review was also conducted for an update on the situation in Europe.

## 2. Results 

### 2.1. A Systematic Review

Publications in the systematic review evaluated antibiotic susceptibility in European populations of *Enterococcus* spp. in 19 studies (Supplementary Table S1), *Staphylococcus* spp. in 14 studies (Supplementary Table S2), and *Streptococcus* spp. in 13 studies (Supplementary Table S3). 

There was a low detection frequency of Gram-positive microorganisms in the reviewed studies (almost always <20%) in comparison to the present findings (27.69%), although the percentage resistance of *Enterococcus* spp. to glycopeptides and ampicillin was higher than in the present series (close to 0%).

### 2.2. Detection and Susceptibility in Our Local Setting

Among the 73,750 urine samples analyzed, SB was identified in 21,838 (29.61%), and antibiograms were performed, revealing gram-positive bacteria in 6047 episodes (27.69%) (Table 1). 

During 2020 and 2021, significant increases were observed in the detection frequency of *E. faecalis* (*p* < 0.001) (from 16.7% in 2019 to 18.89% in 2020 and 20.97% in 2021; Supplementary Figure S2) and *E. faecium* (*p* < 0.001) (from 3.08% in 2019 to 4.85% in 2020 and 5.36% in 2021); (Supplementary Figure S3). Only close-to-significant differences were found in the annual detection frequencies of *Staphylococcus saprophyticus* and *Streptococcus agalactiae* (*p* = 0.05227 and *p* = 0.1541), and there was no change in the frequencies of *S. aureus* (*p* = 0.541) or *S. bovis* (*p* = 0.503).

### 2.3. Enterococcus spp.

*E. faecalis* represented 19.04% of the series and *E. faecium* 3.92% (Table 1). Table 2 exhibits the detection frequency of *Enterococcus* spp. stratified by age, sample origin, and sex. *E. faecalis* was more frequent in under-14-year-olds (23.46% vs. 16.73% in adults) and *E. faecium* in over-65-year-olds (5.22% vs. 3.32% in adults). Both species were more frequent (*p* < 0.001) in hospital than community samples (5% more in the case of *E. faecalis*) and in males than females (8% more in the case of *E. faecalis*).

Supplementary Tables S4 and S5 exhibit changes in antibiotic resistance of the genus *Enterococcus* over the study period. Small and variable but statistically significant differences were observed for the following antibiotics: ampicillin (*p* = 0.02451) in *E. faecalis;* nitrofurantoin (*p* < 0.001) in *E. faecium;* vancomycin (*p* = 0.026), and linezolid (*p* < 0.01) in *E. faeacalis;* and high-level resistance [HLR, given that Enterococcus spp. are intrinsically resistant to aminoglycosides, it is proposed to use the synergistic effect of two antibiotics to achieve the desired bactericide action, one (e.g., beta-lactamases or glycopeptides) acting on the cell wall and the other (aminoglycoside) acting at ribosome level to prevent protein synthesis. The high-level resistance (HLR) test measures the resistance to the aminoglycosides used in this synergy] to streptomycin (*p* < 0.001) and gentamicin (*p* < 0.001) in *E. faecium*. The sole consistent trend over the study period was in the resistance of *E. faecalis* to levofloxacin (from 33.29% in 2016 to 28.02% in 2021, *p* < 0.01; Supplementary Figure S4).

In-vitro results (Supplementary Table S6) revealed statistically significant differences between age groups in the HLR of *E. faecalis* to streptomycin (*p* < 0.001), gentamicin (*p* < 0.001), and the resistance to levofloxacin (*p* < 0.001), by up to 30%, and in the resistance of *E. faecium* to levofloxacin (75% of resistant isolates in under-14-year-olds vs. 94.42% in over-65-year-olds, *p* = 0.02) and nitrofurantoin (0 in under-14-year-olds vs. 12.01% in over-65-year-olds, *p* = 0.01654). Between-sex comparisons showed significantly lower resistance rates of E. *faecalis* to levofloxacin (26.17% in females vs. 36.49% in males, (*p* < 0.001), HLR to streptomycin (30.63% vs. 36.76%, respectively, *p* < 0.001) and gentamicin (34.27% vs. 39.16%, *p* = 0.0011) in females versus males. Samples from females also showed close-to-significantly lower HLR of *E. faecium* to streptomycin (*p* = 0.077) and gentamicin (*p* = 0.2904), with similar quantitative differences as those observed for *E. faecalis*. Comparisons by sample origin found only one statistically significant difference, which was a lower (*p* < 0.001) HLR of *E. faecalis* to gentamicin in community (35.48%) vs. hospital (43.83%) samples.

Supplementary Table S7 displays the susceptibility profile of *Enterococcus* spp. according to EUCAST 2021 and CLSI 2021 criteria in each studied year for the selected antibiotics. No significant differences were found in the resistance of *E. faecalis* and *E. faecium* to ampicillin (*p* = 0.9601 and *p* = 0.973, respectively). When EUCAST criteria were applied, significantly but very slightly higher resistance rates were observed against vancomycin for *E. faecalis* (0.39% vs. 0.19% with CLSI, *p* = 0.02998) and *E*. *faecium* (16.22% vs. 2.70% with CLSI, *p* < 0.0001) and against teicoplanin for *E. faecalis* (0.65% vs. 0.17% with CLSI, *p* < 0.0001).

### 2.4. Staphylococcus spp.

*S. aureus* represented 0.52% of the present series, *S. saprophyticus* 1.13%, and other *Staphylococcus* spp. 0.04% (Table 1). Table 2 exhibits the detection frequency of *Staphylococcus* spp. stratified by sex, age, and sample origin. The detection rate was significantly higher in community *versus* hospital samples for *S. saprophyticus* (1.52% vs. 0.75%, respectively) (*p* < 0.001) but not for *S. aureus* (*p* = 0.533). Detection rates of both species significantly differed between sexes (*p* < 0.001) and age groups (*p* < 0.001). Notably, 96.76% of *S. saprophyticus* isolates were detected in samples from females aged between 14 and 65 years. Only 8 of the 247 isolates studied were from under-14-year-olds and none were from over-65-year-olds.

Supplementary Tables S8 and S9 exhibit the in vitro activity of the antibiotics in isolates of the genus *Staphylococcus* over time. No significant differences were observed over the study period in the percentage resistance of *S. aureus* to oxacillin or cefoxitin (*p* = 0.1949 and *p* = 0.1111, respectively). A significant difference was found in the percentage resistance of penicillin to *S. saprophyticus* (*p* < 0.001) but not to *S. aureus* (*p* = 0.681). No significant difference was found between hospital and community samples (28.85% vs. 26.67%, respectively) in the percentage resistance of *S. aureus* to oxacillin or cefoxitin (*p* = 0.9359 and *p* = 0.856, respectively). The resistance rate to both antibiotics was higher (*p* < 0.05) among over-65-year-olds than among younger patients. No statistically significant difference was found between the sexes in the percentage resistance of *S. aureus* to oxacillin, cefoxitin, or penicillin, although a close-to-significantly higher resistance (*p* = 0.06137) to penicillin was observed in males. The percentage resistance of *S. aureus* to levofloxacin significantly decreased over the study period (*p* = 0.036) and was significantly higher among over-65-year-olds (*p* = 0.04073); however, there was no significant difference between hospital and community samples (chi-square test, *p* = 0.9359) or between the sexes (*p* = 0.391).

There was virtually no resistance of the genus *Staphylococcus* to glycopeptides, finding only one resistant isolate of *S. aureus* and another of *S. saprophyticus*.

Supplementary Table S7 shows the susceptibility profile of *Staphylococcus* spp. for selected antibiotics each year according to EUCAST 2021 and CLSI 2021 criteria. The resistance rate of *S. saprophyticus* isolates to oxacillin was considerably higher (*p* < 0.0001) with CLSI criteria (76.02% to 2.85% with EUCAST), while the resistance rate of *S. aureus* to gentamicin was significantly higher when EUCAST criteria were applied (15.04% vs. 1.77% with CLSI, *p* < 0.0001).

### 2.5. Streptococcus spp.

*S. agalactiae* represents 1.88% of the present series, *Streptococcus* spp. 0.56%, and other *Streptococcus* spp. 0.05% (Table 1). Table 1 shows that the detection frequency of *S. agalactiae* significantly decreased over the past two years (2020 and the first half of 2021) (*p* = 0.042). Table 2 exhibits the detection frequency of *Streptococcus* spp. stratified by sex, age, and sample origin. The detection frequency of *S. agalactiae*, (*p* < 0.001) but not *S. bovis* (*p* = 0.836) was significantly higher among under-65-year-olds (3.26% vs. 0.97%). There was no significant difference in detection frequency of either species between community and hospital samples (*p* = 0.5071 and *p* = 0.713, respectively). However, the detection frequency was significantly higher among females than males for *S. agalactiae* (2.61% vs. 0.77%, *p* < 0.001) and *S. bovis* (0.75% vs. 0.29%, *p* < 0.001).

Supplementary Tables S10 and S11 display the in vitro activity of the different antibiotics against genus *Streptococcus* isolates, showing a significant increase in the percentage resistance of *S. agalactiae* to tetracycline (*p* = 0.02315) (up to 19.31%) but not *S. bovis* (*p* = 0.6124) over the study period. No isolates of this genus were resistant to vancomycin with the application of either CLSI 2021 or EUCAST 2021 breakpoints. No species differed in resistance rate to clindamycin as a function of the year (*p* = 0.219 for *S. agalactiae* and *p* = 0.274 for *S. bovis*). Likewise, the percentage resistance of *S. agalactiae* to clindamycin did not significantly differ between community and hospital samples, age groups, or the sexes (*p* = 0.4696, *p* = 0.1071, and *p* = 0.06137, respectively), although it was close-to-significantly higher among males and under-14-year-olds. Statistical analysis was not possible for the remaining antibiotics due to the very low number of resistant isolates.

Finally, no significant difference in resistance rates of *S. agalactiae* to tetracycline (*p* = 0.5996) or penicillin (*p* = 0.143) was observed between the application of EUCAST and CLSI breakpoints in any year under study (Supplementary Table S7).

## 3. Discussion

This study analyzed a large sample of Gram-positive bacteria obtained in urine cultures. It comprised two parts: a descriptive analysis of clinical, epidemiological, and antibiotic susceptibility data for adults and children with SB at our center over the past five years; and a systematic review of the literature between 2010 and 2021. The review highlighted the scant high-quality studies on Gram-positive resistances in SB, which mostly focus on the most frequent microorganisms (Enterobacteriaceae), with less attention paid to *Enterococcus* spp., *Staphylococcus* spp., and *Streptococcus* spp. A high frequency of SB by *Enterococcus* spp. was detected in the present study (23.13% of 21,838 cases). However, most authors describe the resistance rate of genera but not species [8,9], limiting comparisons with these data.

A wide disparity in detection frequencies and resistances among reviewed articles is largely attributable to differences in study populations. For instance, studies that more frequently reported the isolation of *Enterococcus* spp. were conducted in hospitalized patients [8,10,11] and over-65-year-olds [8]. In this way, infection by *Enterococcus* spp. appears to be more frequent in hospitalized patients, over-65-year-olds, and males, whereas *S. saprophyticus* is detected virtually exclusively in 18- to 65-year-old females, and *S. aureus* is much more frequent among over-65-year-olds and males. Other study populations comprised only females aged between 18 and 65 years with non-complicated UTIs [12] or children [12].

The detection frequency of Gram-positive microorganisms was higher in our hospital series than in most reviewed studies. In comparison to the present findings, only Sánchez García JM et al. [8] and Gajdács M et al. [9] describe comparable detection frequencies of genus *Enterococcus*, only studies in female adults found higher frequencies of genus *Staphylococcus* [13], and only Kornfält Isberg H et al. [14] and Schmiemann G et al. [15], both with small sample sizes, obtained similar frequencies of *Streptococcus* spp. The elevated percentage detection of Gram-positive microorganisms in the present series may be attributable to the large proportion of Enterococci isolates in the pediatric patients (>24% for the genus *Enterococcus*). In contrast, the reviewed articles report a frequency of around 5% in this age group [13,16,17]. This striking difference indicates the need for further research on the possible role of this pathogen in cases of pediatric SB.

Unlike most of the epidemiological studies reviewed, virtually no significant difference in resistance rate was found between community and hospital samples except for a moderately higher resistance of *E. faecalis* to aminoglycosides in hospital samples.

Comparison of resistance rates between the application of EUCAST 2021 and CLSI 2021 criteria yielded only four statistically significant differences (see Results). EUCAST 2021 criteria appear to better detect possible small outbreaks that warn of the possible introduction of strains of glycopeptide-resistant Gram-positive microorganisms, whose percentage detection was generally lower in the present series. Notably, the percentage of resistant *Enterococcus* spp. was lower than in almost all reviewed studies (range, 0.29–0.41%).

The most recent investigations [4] pointed to the pathogenic potential of emerging bacteria described over the past few years (*Aerococcus* spp., *Actinobaculum* spp., *Corynebacterium* spp.), but further follow-up studies are required to fully elucidate their role as pathogenic or contaminant agents [3].

### 3.1. Enterococcus *spp.*

The detection rate of *Enterococcus* spp. differed among the years under study (Table 1), which may reflect differences in the frequency and extent of in-hospital outbreaks or transmissions. A significantly higher percentage of UTIs caused by this genus was observed in elderly, hospitalized, and male patients. Interestingly, although the detection of *Enterococcus* spp. was reduced to minimal rates in 2019, it was significantly increased in 2020 and again in 2021 (Table 1). This may be attributable to the COVID-19 pandemic, given the relationship of these pathogens with hospitalization and catheterization, although resistance rates do not appear to have been affected, except in the case of *E. faecium* to gentamicin. This trend was also observed in other studies during and after the pandemic [18].

In comparison to the present findings, reviewed articles (Supplementary Tables S1–S3) describe a generally higher resistance rate of *E. faecalis* to ampicillin (0.31% vs. rates up to 26.7%), levofloxacin (31.36% vs. rates up to 50%), nitrofurantoin (0.29% vs. rates up to 7.69%), and especially glycopeptides (0.29% vs. rates up to 40%), while they report similar resistance rates to aminoglycosides and fosfomycin.

The development of resistances remained relatively stable over the study period. A significant annual decrease was observed from 2017 in the nitrofurantoin resistance rate of *E. faecium*, in agreement with the report from the EARS-Net [19]. There was also a significant decrease in the levofloxacin-resistance of *E. faecalis*, which may be related to a lesser prescription of quinolones to treat mild UTIs in our setting due to the high resistance of *E. coli* [20,21]. This may also explain the significant increase in the susceptibility of *S. aureus* to levofloxacin over the study period.

One of the treatments for bacteremia and endocarditis caused by *E. faecalis* is ampicillin associated with gentamicin [22,23,24], and the resistance rates of *E. faecalis* to ampicillin and gentamicin were lower in the present series than in the reviewed studies. However, the HLR test result for gentamicin was elevated (36.73%). Accordingly, empirical treatment based on this synergy may be inadequate in around one-third of patients with suspicion of systemic infection by *E. faecalis*. As noted by other authors, account should be taken of this potential drawback in establishing antibiotic protocols for this type of infection, for which the best alternative option might be a combination of ceftriaxone with ampicillin [24,25].

Percentage resistances were higher in males than females against most of the tested antibiotics, highlighting levofloxacin and HLR tests for aminoglycosides in *E. faecalis* and *E. faecium.* Similarly, the resistance rate to most antibiotics (levofloxacin, nitrofurantoin, and HLR tests for aminoglycosides in *E. faecalis* and levofloxacin in *E. faecium*) was significantly higher in over-65-year-olds. However, in some cases (e.g., the HLR test for gentamicin in *E. faecium*) the resistance rate was higher in the pediatric population. This was an unexpected finding.

### 3.2. Staphylococcus *spp.*

The prevalence of MRSA (oxacillin- and cefoxitin-resistance) was lower in the present study than in the reviewed articles. As in the case of the resistance of *E. faecalis* to vancomycin, rigorous surveillance of the resistance of these strains is vital, given their very difficult treatment. No relationship was found between hospitalization and MRSA in the present series. It has been widely reported over the past two decades that the incidence of MRSA is starting to increase in the community [26] and is no longer exclusive to hospital departments. The absence of a significant difference between the present samples of hospital and community origin (28.85% vs. 26.67, respectively) was also described in a recent study.

The less frequent detection of *S. agalactiae* in the urinary tract during 2020 and 2021 may be attributable to the lower number of pregnancies recorded during the SARS-CoV-2 pandemic [27]. It was mostly isolated in samples from young female patients, and there was a non-negligible number of isolates in samples from males (*n* = 68) and over-65-year-olds (*n* = 99), in whom it can also cause disease [28,29,30].

### 3.3. Streptococcus *spp.*

The role of the *S. bovis* group in UTIs is controversial [31,32]. It is mainly observed as a urinary pathogen in patients who are elderly or have comorbidities [33], although there was no statistically significant difference in prevalence between over-65-year-olds (0.53%) and under-15-year-olds (0.72%) in the present series. It may therefore be of interest to conduct further research on the capacity of this *Streptococcus* group to produce disease in the urinary tract of children. The resistance rate of *S. bovis* in our local setting was markedly lower between 2020 and 2021 than between 2012 and 2015 [34], when resistances were higher against all studied antibiotics, with rates of 2% to penicillin, 45% to clindamycin, and 2% to vancomycin. Notwithstanding methodological changes, these data indicate a true decrease in resistances over time.

### 3.4. Limitations

Limitations of this retrospective study include the lack of clinical data on the patients from whom samples were drawn. This information would have provided further insight into the antibiotic resistances observed and might have allowed possible causal relationships to be identified.

## 4. Material and Methods

### 4.1. Systematic Review

The PubMed^®^ MEDLINE database was searched using the following six terms: ”Gram-positive bacteria and urinary tract infection and resistances”; “*Enterococcus* and urinary tract infection and resistances”; “*Staphylococcus* aureus and urinary tract infection and resistances”, “*Staphylococcus saprophyticus* and urinary tract infection and resistances”; “*Streptococcus agalactiae* and urinary tract infection and resistances”; and “*Streptococcus bovis* and resistances”. Inclusion criteria were publication in English or Spanish between 1 January 2010 and 30 June 2021, and the analysis of urine samples. Exclusion criteria were non-European reference population, samples of animal origin, and samples from matrices other than urine. The search retrieved 683, 1128, 1288, 158, 87, and 199 publications, respectively, for each search term. Selection criteria were met by 34 publications, which were included in the review (Supplementary Figure S1).

### 4.2. Detection and Susceptibility in Our Local Setting

A cross-sectional, descriptive, retrospective study was performed of all urine samples with SB received by the Microbiology Laboratory of the Virgen de las Nieves University Hospital in Granada (Spain) between 1 January 2016 and 31 May 2021. No exclusion criteria were applied. Urine culture results were gathered for samples from patients with suspected clinical diagnosis.

In urine samples obtained by spontaneous miction from a permanent catheter, collection bag, or nephrostomy tube, SB was defined by a count of ≥10^5^ CFU (colony forming units)/mL or count of >10^4^ CFU/mL for a single microorganism in the presence of 40 leukocytes/mL in non-centrifuged urine. Urine cultures with the growth of >2 microorganisms were considered contaminated. In urine samples obtained from a temporary urethral catheter, a count of ≥10^4^ CFU/mL for one or two microorganisms was considered significant. All samples were processed according to routine protocols of our clinical microbiology laboratory [35]. Microorganisms grown in habitual culture were identified by means of MALDI-TOF Biotyper (Bruker Daltonics, Billerica, MA, USA) or MicroScan WalkAway (Beckman Coulter, Brea, CA, USA) systems. MicroScan WalkAway was also used for antibiotic susceptibility studies, classifying isolates as susceptible, intermediate, or resistant to each antibiotic according to CLSI recommendations until 2019, and according to EUCAST recommendations from 2020 onwards.

Virgen de las Nieves hospital complex comprises three centers (General Specialty, Mother/Children, and Neurotraumatology). Data on study variables were obtained from the Andalusian Public Health MODULAB ^®^ electronic clinical records system. Samples were categorized by their origin as hospital (specialized care department) or community (outpatient or emergency department, excluding patients hospitalized in previous 48 h). Results were also stratified by patient age (≤14 years, 15–64 years, >65 years) and sex. 

R 4.4.1 software P (https://www.R-project.org/) (accessed on 1 March 2023) was used for data analyses. First, absolute and relative frequencies were calculated for qualitative variables and central tendency and dispersion measures for quantitative variables. The normality of data was verified by the Kolmogorov-Smirnov test. The percentage resistance to different antibiotics in each year was compared with Pearson’s chi-square test or, when applicability conditions were not met (≤20% of expected frequencies < 5), Fisher’s exact test. *p* < 0.05 was considered significant.

The results of applying the distinct criteria were compared by gathering the MIC values of each clinical isolate and applying EUCAST 2021 [36] and CLSI 2021 [37] breakpoint criteria separately to classify the isolate as resistant, intermediate, or susceptible.

**Ethical considerations.** The study protocol complied with the Helsinki Declaration and ethical considerations of epidemiological research [38]. This study was non-interventionist and involved no investigation other than routine procedures. The biological material was solely used for the standard diagnosis of UTI ordered by clinicians. There was no additional sampling or modification of routine diagnosis protocols. Data were analyzed using a fully anonymous database, in which individuals were replaced with infectious episodes (observed at least 6 weeks after any previous episode). The Management Unit of the Clinical Microbiology Department granted permission to access and analyze the data.

## 5. Conclusions

In conclusion, a higher percentage of SB associated with Gram-positive bacteria is observed in our setting than reported in published studies, especially in relation to the genus Enterococcus. In addition, overall resistance rates were stable over time and were higher in males and over-65-year-olds, regardless of the hospital or community origin of the sample. The few significant differences in microbial susceptibility classification between the application of EUCAST 2021 and CLSI 2021 criteria were of little clinical relevance. Nevertheless, EUCAST 2021 guidelines may be more useful for the strict surveillance of small outbreaks of resistance, especially of Enterococcus to glycopeptides. Finally, the results obtained on resistance in our setting suggest that certain empirical treatments should be reconsidered.

## Figures and Tables

**Table 1:** Detection frequency of Gram-positive bacteria in significant bacteriuria in our setting.

Microorganism N (%)	2016	2017	2018	2019	2020	2021	Total
*Actinobaculum* spp.	0	0	2 (0.06) *Actinobaculum* spp. 1 (0.02) *A. schaalii*	1 (0.02) *A. schaalii*	0	0	4 (0.02)
*Aerococcus* spp.	0	2 (0.04)	10 (0.26) *A. urinae* 4 (0.10) *Aerococcus* spp.	6 (0.09) *A. urinae* 1 (0.01) *A. viridans* 4 (0.06) *Aerococcus* spp.	3 (0.08) *Aerococcus* spp. 5 (0.14) *A. urinae* 1 (0.03) *A. viridans*	1 (0.06) *Aerococcus* spp. 2 (0.12) *A. urinae* 2 (0.12) *A. viridans*	41 (0.19)
*Bifidobacterium* spp.	0	0	1 (0.03)	0	2 (0.05)	0	3 (0.01)
*Corynebacterium* spp.	2 (0.05) *C. urealtyticum* 1 (0.02) *C. amycolatum* 1 (0.02) *Corynebacterium* spp.	3 (0.06) *C. urealyticum* 1 (0.02) *C. amycolatum* 1 (0.02) *C. jeikeium*	1 (0.03) *C. jeikeium* 1 (0.03) *C. urealyticum* 1 (0.03) *Corynebacterium* spp.	0	3 (0.08) *C. urealyticum* 2 (0.05) *C. striatum* 1 (0.03) *C. aurimucosum* 1 (0.03) *Corynebacterium* spp.	1 (0.06)	20 (0.09)
*Enterococcus* *faecalis*	761 (19.95)	947 (20.63)	716 (18.58)	678 (16.17)	690 (18.89)	364 (20.97)	4156 (19.04)
*Enterococcus* *faecium*	126 (3.30)	188 (4.09)	142 (3.68)	129 (3.08)	177 (4.85)	93 (5.36)	855 (3.92)
Other *Enterococci* ^1^	7 (0.18)	13 (0.28)	9 (0.23)	2 (0.04)	3 (0.08)	4 (0.23)	38 (0.17)
*Flackamia* spp.	0	0	1 (0.03)	0	0	0	1 (< 0.01)
*Lactobacillus* spp.	2 (0.05) *L. gasseri* 2 (0.05) *L. jensenii* 2 (0.05) *Lactobacillus* spp.	2 (0.05) *L. gasseri* 1 (0.02) *L. fermentum* 1 (0.02) *L. rhamnosus*	2 *Lactobacillus* spp. (0.05)	1 *Lactobacillus* spp. (0.02)	2 (0.05) *Lactobacillus* spp. 1 (0.03) *L. gasseri*	0	16 (0.07)
*S. aureus*	29 (0.76)	22 (0.48)	21 (0.55)	18 (0.43)	12 (0.33)	11 (0.63)	113 (0.52)
*S. saprophyticus*	33 (0.87)	43 (0.94)	55 (1.43)	63 (1.50)	31 (0.85)	22 (1.27)	247 (1.13)
Other *Staphylococci*	1 (0.03) *S. hominis-hominis*	2 (0.04) *S. epidermidis* 1 (0.02) *S. lugdunensis*	1 (0.03) *S. epidermidis* 1 (0.03) *S. lugdunensis*	1 *S. haemolyticus* (0.02)	1 (0.03) *S. epidermidis*	1 (0.06) *S. warneri*	9 (0.04)
*Streptococcus* *agalactiae*	75 (1.97)	94 (2.05)	74 (1.92)	87 (2.07)	59 (1.62)	22 (1.27)	411 (1.88)
*Streptococcus bovis* group	22 (0.58) group *S. bovis*	35 (0.76) group *S. bovis*	2 (0.05) *S. gallolyticus-gallolyticus* 13 (0.34) *S. gallolyticus-pasterianus* 11 (0.28) *S*. *bovis* group	10 (0.23) *S*. *bovis* group 1 (0.02) *S. gallolyticus-gallolyticus* 2 (0.05) *S. gallolyticus-pasterianus*	18 (0.50) *S. bovis* group	8 (0.49) *S. bovis* group 1 (0.06) *S. gallolyticus-pasterianus*	123 (0.56)
Other *Streptococci*	1 (0.03) *S. pyogenes*	3 (0.07) *S. pyogenes* 1 (0.2) *S. mitis*	2 (0.06) *S. salivarius*	1 (0.02) *S. pyogenes*	2 (0.05) *S. pyogenes*	0	10 (0.05)

^1^ We found 10 *E. avium*, 8 *E. durans*, 6 *E. casseliflavus*, 5 *E. gallinarum*, 2 *E. raffinosus*, and 1 *Enterococcus* spp. The species of the remaining 6 *Enterococcus* spp. is not known.

**Table 2:** Detection frequency of *Enterococcus* spp., *Staphylococcus* spp., and *Streptococcus* spp. by patient origin, age, and sex.

*Enterococcus* spp.			*Staphylococcus* spp.		*Streptococcus* spp.		
	*E. faecalis* (%)	*E. faecium* (%)	*S. saprophyticus* (%)	*S. aureus* (%)	*S. agalactiae* (%)	*Streptococcus bovis* Group (%)	Total
**Patient Origin**							
**Community**	1762 (16.44)	225 (2.10)	163 (1.52)	60 (0.56)	214 (1.99)	68 (0.63)	10,717
**Hospitalized**	2381 (21.56)	622 (5.63)	83 (0.75)	52 (0.47)	195 (1.77)	54 (0.49)	11,045
**Age**							
**<14**	656 (23.46)	28 (1)	8 (0.27)	5 (0.18)	24 (0.86)	20 (0.72)	2796
**14–65**	1479 (16.73)	294 (3.32)	239 (2.70)	32 (0.36)	288 (3.26)	49 (0.55)	8843
**>65**	2021 (19.81)	533 (5.22)	0	76 (0.75)	99 (0.97)	54 (0.53)	10,201
**Sex**							
**Females**	2064 (15.77)	479 (3.66)	239 (1.83)	35 (0.27)	342 (2.61)	98 (0.75)	13,081
**Males**	2092 (23.92)	375 (4.29)	8 (0.09)	78 (0.89)	68 (0.77)	25 (0.29)	8744

## Data Availability

The data presented in this study are available in the main text.

## Supplementary Materials

The following supporting information can be downloaded at: https://www.mdpi.com/article/10.3390/antibiotics12071133/s1, Table S1: Detection frequency and percentage resistance of Enterococcus spp. in Europe (2010–2021); Table S2: Detection frequency and percentage resistance of *Staphylococcus* spp. in Europe (2010–2021); Table S3: Detection frequency and percentage resistance of *Streptococcus* spp. in Europe (2010–2021); Table S4: General annualized in vitro resistances (%) of different antibiotics in isolates of E. faecalis; Table S5: General annualized in vitro resistances (%) of different antibiotics in isolates of E. faecium; Table S6: Resistance of *E. faecalis* to levofloxacin and aminoglycosides by patient age; Table S7: Susceptibility profile in isolates adopting EUCAST 2021 and CLSI 2021 criteria; Table S8: General annualized in vitro resistances (%) of different antibiotics in isolates of *S. saprophyticus*; Table S9: General annualized in vitro resistances (%) of different antibiotics in isolates of S. aureus; Table S10: General annualized in vitro resistances (%) of different antibiotics in isolates of *S. agalactiae*; Table S11: General annualized in vitro resistances (%) of different antibiotics in isolates of S. bovis group; Figure S1: Prisma chart for the Systematic Review; Figure S2: Detection frequency (%) of *E. faecalis* between 2016 and 2021; Figure S3: Detection frequency (%) of E. faecium between 2016 and 2021; Figure S4: Resistance (%) of *E. faecalis* to levofloxacin between 2016 and 2021. References [39,40,41,42,43,44,45,46,47,48,49,50,51,52,53,54,55,56] are cited in the Supplementary Materials.
