# Supplementary material for: Antibiotic Resistance Changes in Gram-Positive Bacteria from Urine Cultures: Development Analysis in a Health Area of South-East Spain
Source: Antibiotics (Basel). 2023 Jun 30;12(7):1133. doi: 10.3390/antibiotics12071133 (PMC10376075; doi:10.3390/antibiotics12071133)
Supplement: Supplementary file 1 [file antibiotics-12-01133-s001.zip › antibiotics-2443844-supplementary.pdf]

Supplementary Materials

Table S1. Detection frequency and percentage resistance of Enterococcus spp. in Europe (2010–2021).

Publication	Detection frequency % (n)	Ampicillin	Fosfomycin	Levofloxacin	Ciprofloxacin	Nitrofurantoin	HLR Gentamicin	HLR Streptomycin	Vancomycin	Linezolid
Senel, S et al. (2010 (*E. faecalis*) [16]	6.3% (221)	-							0.90%	
García Viejo, MA et al. (2010) [39] (*Enterococcus* spp.)	% unknown (74)		10.7%	58.6%	48.88%	9.4%				
Palou, Joan et al. (2011) [12] (*E. faecalis*)	3.23% (21)									
Magliano, E. et al. (2012) [40] (*E. faecalis)*	6.3% (868)	3.90%	0%	17.70%		3.90%				
Magliano, E. et al. (2012) [40] (*E. faecium)*	0.08% (11)									
Casal, MM. Et al. (2012) [41] (*E. faecium*)	(170)	60.97% (103)					24.77% (42)	53% (90)	1% (2)	0%
Romero-Cullerés, M. et al. (2012) [42] (*Enterococcus* spp.*)*	13.2% (38)			39%						
Schmiemann, G et al. (2012) [15] (*E. faecalis*)	13.6% (26)		23.08 %		7.69%	7.69 %				
Álvarez-Lerma, F et al. (2013) [43] (*E. faecalis)*	11.09% (281)	0.52% (3)					37.5%		0.44% (1)	
Álvarez-Lerma, F et al. (2013) [43] (*E. faecium)*	2.57% (65)									
Álvarez-Lerma, F et al. (2013) [43] (others *Enterococcus)*	1.62% (41)									
Medina-Polo, J et al. (2015) [44] (*E. faecalis*)	8.4% (30)	26.7%		50%		6.7%	60%			
Aguinaga, A et al. (2018) [45] (*E. faecalis*)	11.1% (5112)		7.4%	32.5% (norfloxacin)		2.8%				
Chervet, D et al. (2018) [46] (*Enterococcus* spp.)	6% (67)									
Benítez-Sala, R. et al (2019) [10] (*Enterococcus* spp.)	17.5% (56)	26.7%		50%			44.1%		3.4%	
Sánchez-García,J.M et al. (2019) [8] (*Enterococcus* spp.)	23.3% (1014)	17.90%	7.2%	55.6%		4%				

Salmanov, AG et al. (2019) [47] (*Enterococcus* spp.)	14.9% (78)						11.3%	
Vazouras, K et al. (2020) [17] (*Enterococcus* spp.)	0.85% (2)							
De Lorenzis, Elisa et al. (2020) [49] (*Enterococcus* spp.)	14.9% (141) *E. faecalis*. 0.5% (5) *E. faecium*	51%			14%		11%	
Kot, Barbara y col. (2021) [11] (*E. faecalis*)	9.3% (15)	7.7%		36.4%		38.5%	0%	
Kot, Barbara y col. (2021) [11] (*E. faecium*)	6.2% (10)	100%		100%		40%	40%	
Gajdács, Márió et al. (2021) [9] (*Enterococcus* spp.)	[1]	C: 1.2%; H: 2.6%	C: 34.1%; H: 39.3%		C: 1.3%; H: 5.8%	C: 29.7%; H: 39.3%	C: 1%; H: 4.6%	C: 0%; H: 0%
Vázquez-Pérez, A. et al. (2021) [13] (*E. faecalis*)	1.8% (3)							

In brackets, the absolute number of strains isolated in the study (n) corresponding to the percentage. HLR: High level of resistance; C: Community; H: Hospital. [1] The detection rate in hospitalized patients was 19.46% (820) for *E. faecalis*, 2.04% (86) for *E. faecium*, and 0.04% (2) for other *Enterococci*. The detection rate in community-origin patients was 19.39% (960) for *E. faecalis*, 0.69% (34) for *E. faecium*, and 0.08% (4) for other *Enterococci*.

Table S2. Detection frequency and percentage resistance of Staphylococcus spp. in Europe (2010–2021).

Publication	Detection Frequency % (n)	Oxacillin	Cefoxitin	Penicillin	Fosfomycin	Nitrofurantoin	Gentamicin	Trimethoprim-sulfamethoxazole	Levofloxacin	Vancomycin	Linezolid
Senel, S et al. (2010) [16] (*S. aureus*)	1.1% (38)			86.8%					8.6%	0%	
Schmiemann, G et al. (2012) [15] (*S. saprophyticus*)	2.1% (4)				100%	0%		0%	0%		
Schmiemann, G et al. (2012) [15] (*S. aureus*)	1.6% (3)				100%	0%		0%	0%		
Schmiemann, G et al. (2012) [15] (other *Staphylococci*)	2.6% (5)				0%	0%		66.67%	0%		
Magliano, E. et al. (2012) [40] (*S. aureus*)	0.4% (51)										
Magliano, E. et al. (2012) [40] (*S. saprophyticus*)	0.15% (21)										
Magliano, E. et al. (2012) [40] (Other *Staphylococci*)	0.05% (7)										
Fabre, R et al. (2013) [49] (*S. saprophyticus*)	1.8% (91)				98.85%	0%	0%	1.1%	0%	0%	
Medina-Polo, J et al. (2015) [44] (*S. aureus*)	1.1% (4)										
Medina-Polo, J et al. (2015) [44] (*S. epidermidis*)	3% (10)										
Adeghate, J et al. (2016) [50] (*S. saprophyticus*)	(66)	0.9%					0.9%	0.9%	0%		
Looney, AT et al. (2017) [51] (*S. aureus*)	0.13% (542)	27.9%	27.9%	27.9%							
Aguinaga, A et al. (2018) [45] (*S. saprophyticus*)	1.6% (737)					0.5%		1%	0.5% (norfloxacin)		
Aguinaga, A et al. (2018) [45] (*S. saprophyticus*)	0.7% (322)				7.4%	0.8%		3.2%	37.1% (norfloxacin)		
Sánchez-García,J.M et al. (2019) [8] (*Staphylococcus* spp.)	0.8% (36)	47.7%				0%	2.8%	0%	27.8%		
Salmanov, AG et al. (2019) [47] (*S. aureus*)	5.4% (28)	39.2%	39.2%								

Salmanov, AG et al. (2019) [51] (*Staphylococcus* coagulase negative)	7.3% (38)	42.1%												3.6%			
Vazouras, K et al. (2020) [17] (*Staphylococcus* spp.)	0.42% (1)																
Kornfalt Isberg, H et al. (2019) [15] (S. saprophyticus)	9% (22)																
Kornfalt Isberg, H et al. (2019) [14] (*Staphylococcus* no *saprophyticus*)	1.23% (3)																
De Lorenzis, Elisa et al. (2020) [48] (*Staphylococcus* spp.)	2% (19) *S. aureus* 4.7% (45) *Staphylococcus* coagulase-negative	61%								24%				0%			
Gajdács, Márió et al. (2021) [9] (*Staphylococcus* sp.)	[1]		C	H		C	H	C	H	C	H	C	H	C	H	C	H
			15.6%	35.8%		0%	6.3%	3.4%	10.5%	16.9%	20%	44.1%	77.9%	0%	0%	0%	0%

[1] The detection rate in hospitalized patients was 1.68% (71) for *S. aureus*, 0.07% (3) for *S. saprophyticus*, and 0.30% (13) for other *Staphylococci*. The detection rate in community-origin patients was 1.31% (65) for *S. aureus*, 0.14% (7) for *S. saprophyticus*, and 0.10% (5) for other *Staphylococci*.

Table S3. Detection frequency and percentage resistance of Streptococcus spp. in Europe (2010–2021).

**Publication**	**Detection frequency % (n)**	**Penicillin**	**Fosfomycin**	**Clindamycin**	**Trimethoprim-sulfamethoxazole**	**Tetracycline**	**Levofloxacin**	**Vancomycin**	**Linezolid**
Schmiemann, G et al. (2012) [15] (*S. agalactie*)	4.2% (8)		0%		37.5%		25%		
Matesanz, M et al. (2014) [31] (*S. bovis* group)	(152)	0%	23%						
Matani, C et al. (2016) [52] (*S.agalactiae*)	(65)	12.07%			4.62%	15.62%	9.23%	7.81%	0%
Gómez-Camarasa, C et al. (2016) [34] (*S.* group *bovis*)	0.5% (91)	2%		45%			30%	2%	1%
Aguinaga, A et al. (2018) [45] (*S. agalactiae)*	2.2% (1013)	0% (amoxicillin)	13.4%						
López, Y et al. (2018) [53] (*S. agalactiae*)	(242)	0%		17.4%				0%	
Kornfalt Isberg, H et al. (2019) [14] (*S.agalactie*)	4% (10)								
Sánchez-García,J.M et al. (2019) [8] (*Streptococcus* spp.)	1.9% (83)		1.2%		0%		2.5%		
Rigvava, S et al. (2019) [54] (*S. agalactiae*)	(39)	0%		12%				0%	
Vazouras, K et al. (2020) [17] (*Streptococcus* sp.)	0.42% (1)								
De Lorenzis, Elisa et al. (2020) [48] (*Streptococcus* spp.)	1% (10) *S. agalactie*. 0.8% (8) other *Streptococci*				66.67%			0%	
Genovese, C et al. (2020) [55] (*S. agalactiae*)	(3494)	0.2%		31.2%			4.6%	0%	
Gergova, RT et al. (2021) [56] (*S.agalactiae*)	(792)	0%		15.89%		94.62%	10.28%		

Table S4. General annualized *in vitro* resistances (%) of different antibiotics in isolates of *E. faecalis*.

Antibiotic	2016	2017	2018	2019	2020	2021	Total
Ampicillin	4 (0.53)	1 (0.11)	3 (0.42)	0	1 (0.15)	4 (1.10)	13 (0.31)
HLR Streptomycin	258 (34.04)	327 (34.57)	232 (32.54)	227 (33.53)	234 (34.26)	118 (32.42)	1396 (33.71)
Gentamicin	302 (39.84)	347 (36.68)	270 (37.87)	237 (35.01)	233 (34.11)	132 (36.26)	1521 (36.73)
Fosfomycin	31 (4.07)	21 (2.22)	27 (3.77)	22 (3.24)	28 (4.06)	12 (3.30)	141 (3.39)
Vancomycin	0	1 (0.11)	5 (0.70)	1 (0.15)	1 (0.4)	3 (0.83)	12 (0.29)
Teicoplanin	1 (0.13)	1 (0.11)	3 (0.42)	1 (0.15)	2 (0.29)	3 (0.83)	11 (0.31)
Linezolid	2 (0.27)	2 (0.21)	13 (1.83)	11 (1.62)	7 (1.02)	5 (1.38)	40 (0.97)
Nitrofurantoin	5 (0.66)	0	0	2 (0.29)	5 (0.73)	0	12 (0.29)
Levofloxacin	253 (33.29)	306 (32.31)	240 (33.52)	208 (30.68)	194 (28.16)	102 (28.02)	1303 (31.36)

Table S5. General annualized *in vitro* resistances (%) of different antibiotics in isolates of *E. faecium*.

Antibiotic	2016	2017	2018	2019	2020	2021	Total
Ampicillin	122 (96.83)	175 (94.09)	132 (92.96)	124 (96.12)	163 (92.61)	92 (98.92)	808 (94.84)
HLR Streptomycin	99 (78.57)	79 (42.47)	81 (57.45)	89 (68.99)	129 (73.30)	68 (73.12)	545 (64.04)
HLR Gentamicin	51 (40.48)	94 (50.54)	54 (38.30)	34 (26.36)	66 (37.50)	54 (41.94)	353 (41.55)
Fosfomycin	6 (4.76)	13 (6.91)	17 (12.06)	11 (8.53)	19 (10.73)	5 (5.38)	71 (8.31)
Vancomycin	0	1 (0.53)	2 (1.41)	0	0	0	3 (0.35)
Teicoplanin	0	1 (0.53)	3 (2.13)	0	0	0	4 (0.47)
Linezolid	0	1 (0.53)	1 (0.71)	0	5 (2.82)	1 (1.08)	8 (0.94)
Nitrofurantoin	7 (5.56)	31 (16.49)	22 (15.49)	11 (8.73)	11 (6,.9)	4 (4.40)	86 (10.18)
Levofloxacin	119 (94.44)	172 (91.49)	131 (92.25)	120 (94.49)	162 (91.53)	91 (97.85)	795 (93.09)

Table S6. Resistance of *E. faecalis* to levofloxacin and aminoglycosides by patient age.

Age	R Levofloxacin (%)	HLR (%) Gentamicin	HLR (%) Streptomycin	Total
< 14 a.	26 (3.97)	133 (20.18)	111 (16.97)	654
Adults	398 (26.91)	490 (33.26)	443 (30.57)	1473
> 65 a	899 (39.77)	899 (44.64)	842 (41.81)	2015

R: resistant.

Table S7. Susceptibility profile in isolates adopting EUCAST 2021 and CLSI 2021 criteria.

		E. faecalis				*E. faecium*				Other *Enterococci*			
**Antibiotic**	**Criteria**	**I (%)**	**R (%)**	**S (%)**	**Total**	**I (%)**	**R (%)**	**S (%)**	**Total**	**I (%)**	**R (%)**	**S (%)**	**Total**
	EUCAST	14 (0.34)	13 (0.31)	4113 (99.35)		2 (0.23)	806 (94.71)	43 (5.05)		0	9 (24.32)	28 (75.67)	
Ampicillin	CLSI	13 (12*)	12*	4123 (99.59)	4143	807 (805*)	805*	45 (5.29)	851	9*	9*	28	37
	EUCAST	0	12 (0.29)	4138 (99.59)		0	86 (10.19)	758 (89.81)		0	0	37 (100)	
Nitrofurantoin	CLSI	35 (12*)	12*	4125 (99.16)	4156	277 (86*)	86*	567 (67.18)	844	2 (5.41)	0	35 (94.59)	37
	EUCAST	0	1302 (31.36)	2850 (68.64)		0	794 (93.19)	58 (6.81)		0	11 (29.73)	26 (70.27)	
Levofloxacin	CLSI	1331 (1302*)	1302*	2821 (67.94)	4156	808 (794*)	794*	44 (5.16)	852	12 (9*)	10 (9*)	24 (64.86)	37
	EUCAST	0	16 (0.39)	4134 (99.61)		0	5 (0.59)	850 (99.41)		0	6 (16.22)	31 (83.78)	
Vancomycin	CLSI	14 (6*)	8 (6*)	4134 (99.61)	4150	5 (3*)	3*	850 (99.41)	855	6 (1*)	1*	31 (83.78)	37
	EUCAST	0	27 (0.65)	4116 (99.35)		0	6 (070)	846 (99.30)		0	1 (2.70)	36 (97.30)	
Teicoplanin	CLSI	8 (7*)	7*	4134 (99.81)	4143	0	4 (0.47)	848 (99.53)	852	1*	1*	36 (97.30)	37
	EUCAST	0	41 (1.00)	4087 (99.00)		0	8 (0.94)	843 (99.06)		0	2 (5.40)	35 (94.60)	
Linezolid	CLSI	34 (0.82)	39 (0.94)	4055 (98.23)	4128	12 (1.41)	8 (0.94)	831 (97.65)	851	0	2 (5.40)	35 (94.60)	37

		S. saprophyticus				*S. aureus*			
		**I (%)**	**R (%)**	**S (%)**	**Total**	**I (%)**	**R (%)**	**S (%)**	**Total**
	EUCAST	0	7 (2.85)	239 (97.15)		0	31 (31.67)	82 (68.33)	
Oxacillin	CLSI	0	187 (76.02)	59 (23.98)	246	31*	31*	82 (68.33)	113
	EUCAST	7*	7*	239 (97.15)		0	32 (28.22)	81 (71.68)	
Cefoxitin	CLSI	NC	NC	NC	246	32*	32*	81 (71.68)	113
	EUCAST	NC	NC	NC		0	99 (87.61)	14 (12.39)	
Penicillin	CLSI	1 (0.41)	54 (21.95)	191 (77.64)	246	0	99 (87.61)	14 (12.39)	113
	EUCAST	0	2 (0.81)	245 (99.19)		0	17 (15.04)	96 (84.96)	
Gentamicin	CLSI	1 (0.41)	0	246 (99.59)	247	0	2 (1.77)	111 (98.23)	113
	EUCAST	0	2 (0.82)	244 (99.18)		0	1 (0.88)	112 (99.12)	
Vancomycin	CLSI	1 (0.41)	1 (0.41)	244 (99.18)	246	1 (0.88)	0	112 (99.12)	113
Teicoplanin	EUCAST	0	1 (0.41)	245 (99.59)		0	1 (0.88)	112 (99.12)	
	CLSI	0	1 (0.41)	245 (99.59)	246	0	0	113 (100)	113

		S. agalactiae				*S. bovis* group			
		**I (%)**	**R (%)**	**S (%)**	**Total**	**I (%)**	**R (%)**	**S (%)**	**Total**
	EUCAST	0	3 (0.75)	399 (99.25)		4 (3.25)	1 (0.81)	118 (95.93)	
Penicillin	CLSI	0	9 (2.24)	393 (97.76)	402	3 (2.44)	1 (0.81)	119 (96.74)	123
	EUCAST	28 (6.81)	15 (3.65)	368 (89.54)		NC	NC	NC	
Levofloxacin	CLSI	0	15 (3.65)	396 (96.35)	411	1 (0.86)	10 (8.62)	105 (90.52)	116
	EUCAST	3 (0.74)	340 (83.54)	64 (15.72)		NC	NC	NC	
Tetracycline	CLSI	6 (1.47)	334 (82.06)	67 (16.46)	407	2 (1.69)	95 (80.51)	21 (17.80)	118
	EUCAST	0	86 (21.18)	320 (78.82)		0	30 (25.42)	88 (74.57)	118

Clindamycin	CLSI	7 (1.72)	86 (21.18)	313 (77.09)	406	3 (2.54)	30 (25.42)	85 (72.03)	
	EUCAST	0	3 (0.75)	400 (99.25)		0	0	116 (100)	
Linezolid	CLSI	1 (0.25)	2 (0.50)	407 (100)	403	0	0	116 (100)	116

The numbers in italic and with asterisk (n*) indicate the number of microorganisms classified by both sets of criteria as intermediate or resistant

NC: Non-calculable due to lack of established breakpoint.

In some cases, the MIC value reported by laboratories does not allow an accurate determination of the resistant or intermediate nature of the microorganism, given the utilization of different criteria and breakpoints. For instance, if an isolate is given a MIC >2 mg/L, and the value to classify it as intermediate is >2 mg/L and the value to classify it as resistant is >4 mg/L, it could be classified as intermediate or resistant, and a more precise MIC value is needed to determine its classification with certainty. For this reason, the percentages in parentheses do not add up to 100% because some are classified as both resistant and intermediate.

Table S8. General annualized *in vitro* resistances (%) of different antibiotics in isolates of *S. saprophyticus*.

**Antibiotic**	**2016**	**2017**	**2018**	**2019**	**2020**	**2021**	**Total**
Trimethoprim-sulfamethoxazole	0	0	0	0	0	0	0
Fosfomycin	0	1 (2.86)	0	0	0	0	1 (0.83)
Tetracycline	13 (76.47)	26 (74.29)	20 (76.92)	12 (92.31)	15 (83.33)	9 (100)	95 (80.51)
Penicillin	0	0	0	0	0	1 (11.11)	1 (0.81)
Clindamycin	4 (23.53)	16 (45.71)	3 (11.54)	3 (23.08)	2 (11.11)	2 (22.22)	30 (25.42)
Levofloxacin	0	4 (11.43)	3 (11.54)	1 (7.69)	1 (5.56)	1 (11.11)	10 (8.62)
Vancomycin	0	0	0	0	0	0	0
Linezolid	0	0	0	0	0	0	0

Table S9. General annualized *in vitro* resistances (%) of different antibiotics in isolates of *S. aureus*.

Antibiotic	2016	2017	2018	2019	2020	2021	Total
Oxacillin	7 (24.14)	10 (45.45)	4 (19.05)	4 (22.22)	5 (41.67)	1 (9.09)	31 (27.43)
Cefoxitin	7 (24.14)	10 (45.45)	4 (19.05)	4 (22.22)	6 (50)	1 (9.09)	32 (28.22)
Penicillin	26 (89.66)	21 (95.45)	17 (80.95)	16 (88.89)	10 (83.33)	9 (81.82)	99 (87.61)
Gentamicin	0	2 (9.09)	0	2 (11.11)	2 (16.67)	1 (9.09)	7 (6.19)
Trimethoprim-sulfamethoxazole	0	0	0	0	0	0	0
Nitrofurantoin	0	0	0	0	0	0	0
Levofloxacin	8 (27.59)	12 (54.55)	5 (23.81)	3 (16.67)	3 (25)	2 (18.18)	33 (29.20)
Vancomycin	0	0	0	0	0	0	0
Teicoplanin	0	0	0	0	1 (8.33)	0	1 (0.88)
Fosfomycin	1 (3.45)	4 (18.18)	1 (4.76)	0	1 (8.33)	0	7 (6.19)

Table S10. General annualized *in vitro* resistances (%) of different antibiotics in isolates of *S. agalactiae*.

Antibiotic	2016	2017	2018	2019	2020	2021	Total
Oxacillin	1 (3.03)	1 (2.33)	1 (1.82)	4 (6.45)	0	1 (4.55)	8 (3.25)
Cefoxitin	1 (3.03)	1 (2.33)	1 (1.82)	2 (3.17)	0	2 (9.09)	7 (2.85)
Penicillin	8 (24.24)	15 (34.88)	12 (21.82)	8 (12.70)	9 (29.03)	3 (13.64)	55 (22.36)
Gentamicin	0	0	0	0	0	1 (4.55)	1 (0.41)
Trimethoprim-sulfamethoxazole	0	0	0	1 (1.59)	0	1 (4.55)	2 (0.81)
Nitrofurantoin	0	0	0	0	0	0	0
Levofloxacin	0	1 (2.33)	0	0	0	1 (4.55)	2 (0.81)
Vancomycin	0	0	0	1 (1.61)	0	0	1 (0.41)
Teicoplanin	0	0	0	1 (1.61)	0	0	1 (0.41)
Fosfomycin	0	0	0	0	0	0	0

Table S11. General annualized *in vitro* resistances (%) of different antibiotics in isolates of *S. bovis* group.

Antibiotic	2016	2017	2018	2019	2020	2021	Total
Trimethoprim-sulfamethoxazole	0	0	0	1 (1.15)	1 (1.69)	0	2 (0.50)
Fosfomycin	2 (2.67)	3 (3.19)	1 (1.35)	5 (5.75)	0	0	11 (2.68)
Tetracycline	59 (79.73)	70 (76.09)	59 (79.73)	83 (95.40)	47 (81.03)	19 (86.36)	337 (82.80)
Penicillin	0	0	0	3 (3.49)	0	0	3 (0.75)

Clindamycin	14 (18.92)	11 (12.09)	21 (28.38)	23 (26.44)	9 (15.52)	8 (36.36)	86 (21.18)
Levofloxacin	0	4 (4.26)	3 (4.05)	4 (4.60)	4 (6.78)	0	15 (3.65)
Vancomycin	0	0	0	0	0	0	0
Linezolid	0	0	0	0	1 (1.72)	0	1 (0.25)

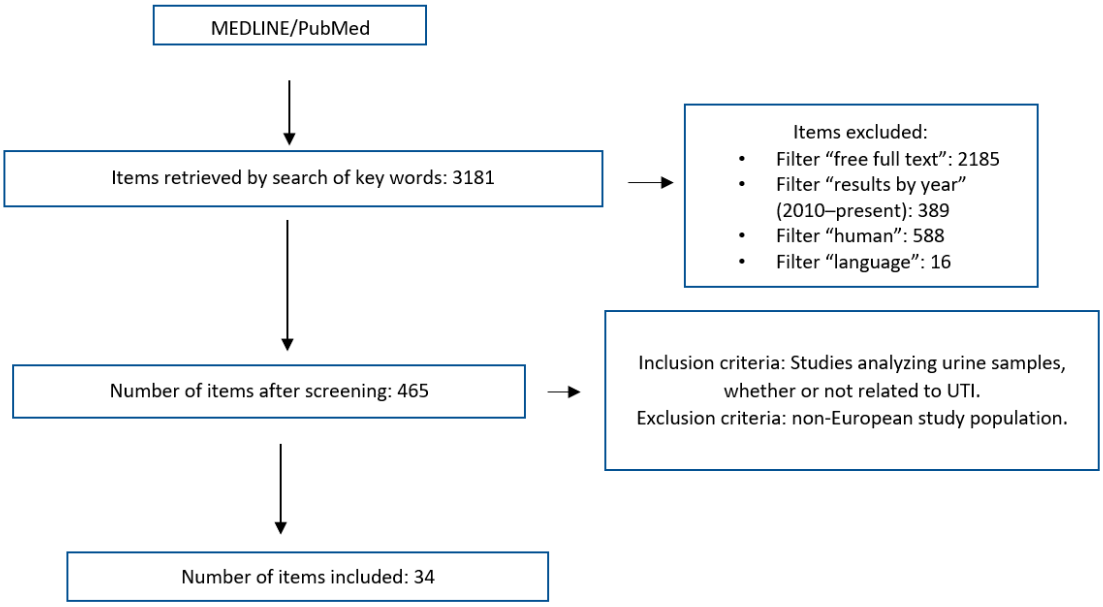

Figure S1. Prisma chart for the Systematic Review.

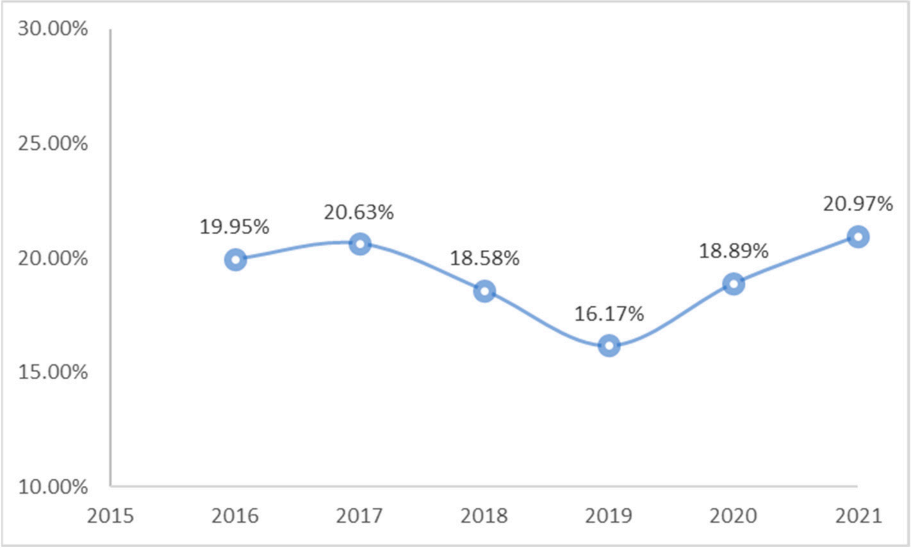

Figure S2. Detection frequency (%) of *E. faecalis* between 2016 and 2021.

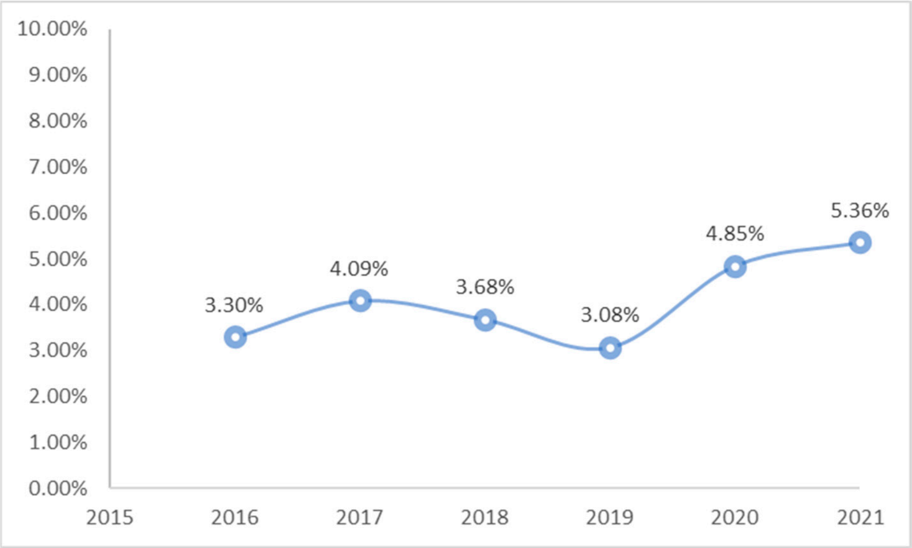

Figure S3. Detection frequency (%) of *E. faecium* between 2016 and 2021.

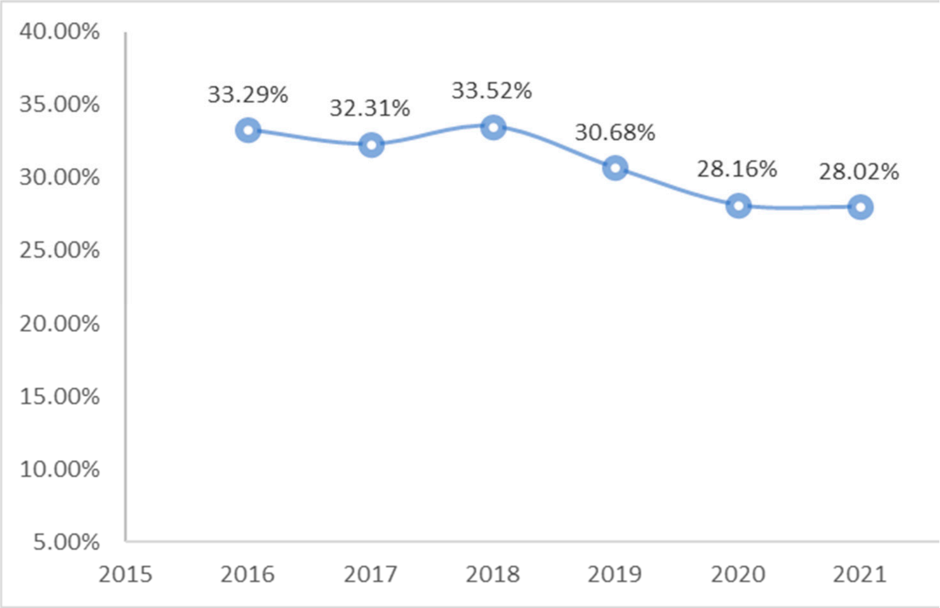

Figure S4. Resistance (%) of *E. faecalis* to levofloxacin between 2016 and 2021.
